# A Pediatric Case of Systemic Lupus Erythematosus Developed 10 Years after Cord Blood Transplantation for Juvenile Myelomonocytic Leukemia

**DOI:** 10.1155/2012/619126

**Published:** 2012-12-10

**Authors:** Masayuki Nagasawa, Yuki Aoki

**Affiliations:** Department of Developmental Biology, Post Graduate School, Tokyo Medical and Dental University, 5–45, Yushima 1-chome, Bunkyo-ku, Tokyo 113-8519, Japan

## Abstract

Allogeneic hematopoietic stem cell transplantation (allo-HSCT) is a most powerful immunotherapy for hematological malignancies. However, the impact of immunological disturbances as a result of allo-HSCT is not understood well. We experienced an 11-year-old boy who presented with systemic lupus erythemathosus (SLE) 10 years after unrelated cord blood transplantation of male origin for juvenile myelomonocytic leukemia (JMML) with monosomy 7. Bone marrow examination showed complete remission without monosomy 7. Genetic analysis of peripheral blood revealed mixed chimera with recipient cells consisting of <5% of T cells, 50–60% of B cells, 60–75% of NK cells, 70–80% of macrophages, and 50–60% of granulocytes. Significance of persistent mixed chimera as a cause of SLE is discussed.

## 1. Introduction

Allogeneic hematopoietic stem cell transplantation (allo-HSCT) is an effective treatment to cure the hematological malignancy through the alloimmune reaction. However, excessive alloimmune reaction triggers acute graft versus host disease (aGVHD) and it attacks various organs and induces their dysfunction, which is often life-threatening. Chronic GVHD is based on more insidious alloimmune reaction and its clinical symptoms sometimes mimic autoimmune diseases. In allo-HSCT, donor cells usually replace the bone marrow cells completely, which is called complete chimera. Donor and recipient hematopoietic cells sometimes coexist stably, which is called mixed chimera. Although mixed chimera is a sign of relapse of hematological malignancy, there are cases of stable and persistent chimera without relapse. However, the impact of immunological disturbances as a result of allo-HSCT and mixed chimera is not understood well. We had an 11-year-old boy who presented with SLE 10 years after allogeneic cord blood transplantation. Interestingly, most of T cells were of donor origin, but B cells, NK cells, macrophages, and granulocytes were mixed chimera. 

## 2. Case Report

At 6-month-old, he presented with hepatosplenomegaly and skin eruption. Bone marrow examination revealed monosomy 7 and he was diagnosed as juvenile myelomonocytic leukemia through clinical evaluation. At 15-month-old, he received HLA-DRB1 one locus mismatched male-derived cord blood transplantation. Conditioning regimen was myeloablative (busulfan (140 mg/m^2^  × 4) + etoposide (15 mg /kg/day × 4) + cyclophosphamide (60 mg/kg × 2) + antithymocyte globulin (2.5 mg/kg × 4)). GVHD prophylaxis was cyclosporine and short-term methotrexate. SCT was successful with only mild GVHD. Complete chimera was determined on day 41. He has been free of immunosuppressant 8 months after SCT. 

10 years after SCT, he complained of wrist and ankle pain. Blood examination revealed marked thrombocytopenia (1 × 10^4^/*μ*L) and he was admitted for further examination. The hemogram at the regular checkup 8 months before admission was normal. He was 164.5 cm and 54.7 kg. Physical examination was unremarkable except for dispersed petechie on face, oral cavity, and extremities and mild swelling of both wrists. The hemogram showed white blood cell of 2100/*μ*L, granulocyte of 168/*μ*L, hemoglobin of 12.1 g/dL, platelet of  0.3 × 10^4^/*μ*L, and reticulocyte of 7.6 × 10^4^/*μ*L. Serological examinations were C3 of 54 mg/dL (74–130), C4 of 13 mg/dL (11–30), CH50 of 26 U/mL (26–49), IgG of 1800 mg/dL, IgM of 917 mg/dL, IgA of 318 mg/dL, sIL-2R of 2720 U/mL (0–550), antinuclear antibody of x1280, anti-DNA antibody of 37 IU/mL (0–6), anti-dsDNA antibody of 157 IU/mL (0–12), anti-ssDNA antibody of 269 U/mL (0–25), anti-SS-A antibody of 7.0 U/mL (0–10), anti-SS-B antibody of 7.0 U/mL (0–10), anti-Sm antibody of 7.0 U/mL (0–10), and anti-RNP antibody of 7.0 U/mL (0–10) (number in the parenthesis means the normal value). Antiplatelet antibody, antineutrophil antibody, and direct Coombs test were positive. Bone marrow examination presented normocellular marrow with normal number of megakaryocytes, normal karyotype, and no atypical cells. FISH analysis detected no monosomy 7 cells in bone marrow and peripheral blood. 

FCM analysis of peripheral lymphocytes revealed that CD3: 79.6%, CD4: 38.1%, CD8: 36.1%, CD19: 9.6%, and CD56: 2.7%. CD4+CD25+ cells comprised 9.8% of CD4+T cells.

He was diagnosed as systemic lupus erythematosus according to the international diagnostic criteria, and prednisolone (PSL; 40 mg/day) was started with the combination of high-dose *γ*-globulin (1 g/kg) for thrombocytopenia. Clinical response was rapid and remarkable. Lumbar puncture performed before the start of PSL showed no abnormal findings. Urinalysis was normal, and renal biopsy performed after hematological remission presented no significant histological findings.

**Figure 1:**
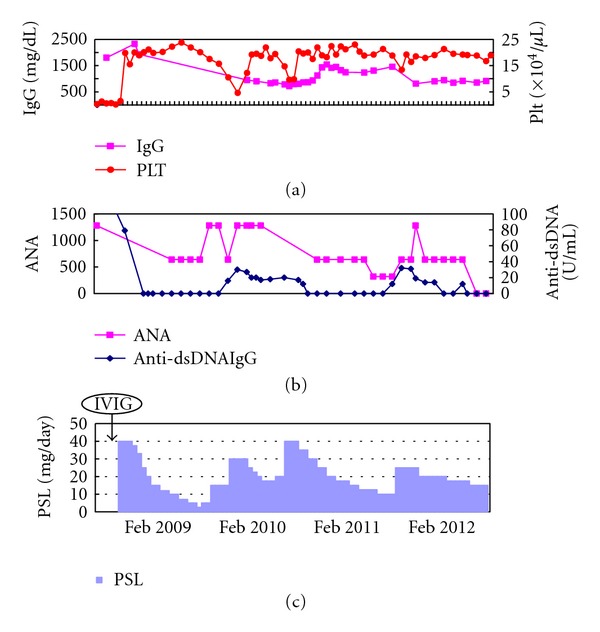
Clinical course of the patient.

After 4 weeks, PSL was gradually tapered, and 8 month later, PSL was tapered as low as to 5 mg/day, when thrombocytopenia recurred. Responsiveness to PSL was as well as at the initial treatment. Although a few serological relapse occurred along with the reduction of PSL, he has been clinically and serologically remission on 10–15 mg of daily PSL (Figure 1). 

## 3. Chimerism Analysis

Chimerism analysis was performed by comparing the three different PCR products of microsatellite regions (D9S304, D21S1437, and D8S1179) which discriminate recipient from donor. The cells from the buccal membrane and nails were used as recipient cells. Granulocytes were obtained by depleting the mononuclear cells by gradient-based centrifugation method. T cells, B cells, NK cells, and macrophages were positively selected from the mononuclear cells by using anti-CD3, anti-CD19, anti-CD56, and anti-CD14 antibody conjugated immunomagnetic beads. 

**Figure 2:**
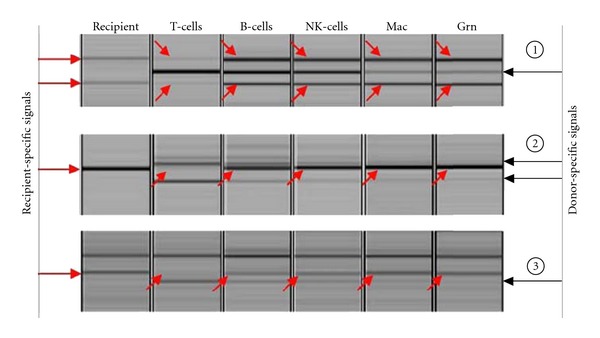
Chimerism analysis of the patient. DNA of each microsatellite region (*➀* D9S304, *➁* D21S1437, *➂* D8S1179) was amplified by PCR and the expanded DNA was separated by electorophoresis. Arrows indicate the recipient- or donor-specific bands. The data showed the result performed in Aug 2012.

At the onset of SLE, recipient cells consisted of <5% of T cells, 50–60% of B cells, 60–75% of NK cells, 70–80% of macrophages, and 50–60% of granulocytes. 8 months later, recipient cells consisted of 40–50% of white blood cells, 10–20% of mononuclear cells, and 70–80% of granulocytes. 3 year later, when he was in clinically and serologically remission on 12.5 mg of daily prednisolone, recipient cells still consisted of <10% of T cells, 50–60% of B cells, 65–75% of NK cells, 85–90% of macrophages, and 85–95% of granulocytes (Figure 2).

## 4. Discussion

JMML is a hematological malignancy which is resistant to chemotherapy, and allo-SCT is recommended to cure [1, 2]. Concerning with the pathogenesis, genetic disturbances of RAS signaling pathway has been disclosed recently [2, 3]. It has been reported that early mixed chimera is a warning of relapse in JMML [4]. However, a few cases have been reported to keep long-term remission with the persistent mixed chimera after allogeneic cord blood transplantation [5]. In some cases of JMML, autoantibody production has been also reported [6]. At the onset of SLE in this patient, relapse of JMML was excluded. Furthermore, mutation of RAS and PTPN11 was not found in this patient at the onset of SLE (data not shown).

SLE is a complex inflammatory disease which involves various organs. The incidence is reported to be 10–150 cases per 100,000 population, and the women of childbearing age are mostly affected. The incidence of pediatric SLE is much less and it occurs rarely less than 5-years old. The genetic, environmental, and hormonal factors are considered to be involved in the pathogenesis of SLE. Although, the recent advances of molecular biology have disclosed the genetic background of SLE in detail, genetic involvements are multifactorial and complicated [7, 8]. Generally speaking, pediatric SLE is more active and severe than adult SLE. Furthermore, involvement of kidney and CNS is more frequently observed than adult SLE [9]. CNS and kidney were intact in our case, and responsiveness to steroid was remarkable. In this sense, our case is clinically atypical as a pediatric SLE.

From the immunological point of views, the basic pathogenesis of SLE is a disturbance of immune regulations, which induces abnormal autoimmune responses. It is well known that autoimmune diseases are occasionally complicated in the patients with persistent chronic GVHD [10]. But SLE after SCT has been rarely reported in the literature. It has been reported that autoimmune hemolytic anemia and cytopenias are observed in very young infants who received unrelated cord blood transplantation. In their report, most of the autoimmune cytopenias occurred within two years after cord blood transplantation [11]. There are rare case reports of patients developing autoimmune cytopenia from 6–9 years after cardiac transplantation, which requires life-long usage of calcineurin inhibitor [12]. It is postulated that treatment of long-term calcineurin inhibitors may affect the developing thymus or function of regulatory T cells, resulting in the development of autoimmune diseases [13]. In our patient, calcineurin inhibitor was used for only 8 months. 

On the other hand, it has been reported that autoimmune diseases are frequently found in Wiskott-Aldrich syndrome patients with persistent mixed chimera after allo-HSCT even in the absence of chronic GVHD [14]. It is speculated that residual recipient cells are important in the pathogenesis of autoimmune diseases after allo-HSCT, because it is known that patients with Wiskott-Aldrich syndrome themselves are subject to autoimmune diseases [15]. Perruche et al. have reported that anti-dsDNA antibody production is frequently observed in the persistent mixed chimera mice model induced by reduced-intensity conditioning [16]. They have concluded that residual host-derived B cells but not dendritic cells are important in the production of autoantibody. In our patient, majority of T cells are of donor origin, but half of B cells are of recipient origin, which is quite similar to mixed chimera mice model by Saas et al. Considering the age, sex, and family history, susceptibility to SLE seems acquired, and persistent mixed chimera may be strongly related with the pathogenesis of SLE in this patient.

It is not clear how long mixed chimera has continued in this patient. He was hematologically normal just 8 months before the diagnosis of SLE and the appearance of petechie was relatively acute-onset. However, it seems that mixed chimera is stable from the persistence of mixed chimera for at least three years even in the remission of SLE.

We would like to emphasize the clinical impact of persistent mixed chimera after SCT on the immune development especially in very younger patients. It is required to follow the chimerism status of patients who received allo-HSCT for long period to elucidate the impact of allo-HSCT on the immune development or disturbance in younger children.

This case is also unique and valuable to consider the pathogenesis of SLE or autoimmune diseases.

## Abbreviations

SLE: Systemic lupus erythematosus
JMML: Juvenile myelomonocytic leukemia
allo-HSCT: Allogeneic hematopoietic stem cell transplantation
HLA: Human leukocyte antigen
GVHD: Graft versus host disease
FISH: Fluorescence in situ hybridization
PSL: Prednisolone
CNS: Central nervous system
FCM: Flow cytometory.
